# Functioning in an Illness and Quality of Life versus the Prevalence of Depression and Anxiety Disorders in Patients with High Cardiovascular Risk

**DOI:** 10.3390/nursrep14030191

**Published:** 2024-09-23

**Authors:** Piotr Michalski, Agata Kosobucka-Ozdoba, Łukasz Pietrzykowski, Michał Kasprzak, Klaudyna Grzelakowska, Alicja Rzepka-Cholasińska, Aldona Kubica

**Affiliations:** 1 Department of Cardiac Rehabilitation and Health Promotion, Collegium Medicum in Bydgoszcz, Nicolaus Copernicus University in Torun, Marii Sklodowskiej-Curie St. 9, 85-094 Bydgoszcz, Poland; michalski.piotr@onet.eu (P.M.); akosobucka@wp.pl (A.K.-O.); alicja_rzepka@vp.pl (A.R.-C.); aldona.kubica@gmail.com (A.K.); 2 Department of Cardiology and Internal Medicine, Collegium Medicum in Bydgoszcz, Nicolaus Copernicus University in Torun, Marii Sklodowskiej-Curie St. 9, 85-094 Bydgoszcz, Poland; medkas@o2.pl (M.K.); klaudyna.grzelakowska@gmail.com (K.G.)

**Keywords:** chronic disease, quality of life, depression, anxiety

## Abstract

Background: A chronic disease occurring in a person’s life is a stressor, disrupting every aspect of their life. Objectives: This study aims to assess the relationship between functioning in chronic illness and quality of life with the prevalence of symptoms of depression and anxiety in patients with high cardiovascular risk. Material and methods: This study included 200 patients (aged 18–80 years) under the care of a primary care physician, diagnosed with hypertension and/or hypercholesterolemia, and/or diabetes between 6 and 24 months before the enrollment. The presented analysis assessed the symptoms of anxiety and depression using the Hospital Anxiety and Depression Scale (HADS); and the quality of life of patients with cardiovascular disease using the Heart Quality of Life (HeartQoL) questionnaire and functioning in chronic illness using the Functioning in Chronic Illness Scale (FCIS). Results: The HADS scores amounted to 4.34 ± 3.414 points for the HADS-Anxiety subscale and 3.20 ± 2.979 points for the HADS-Depression subscale. The score indicative of functioning in chronic illness assessed with the FCIS was 98.32 ± 13.89 points. The independent predictors of HADS-anxiety were HeartQoL Emotional and FCIS Global, while HeartQoL Global and FCIS Global were the independent predictors for HADS-depression. Better functioning in chronic illness (FCIS Global) was associated with less frequent symptoms of anxiety and depression based on the HADS: HADS-Anxiety (R Spearmann = −0.3969; *p* < 0.0001) and HADS-Depression (R Spearmann = −0.5884; *p* < 0.0001). Higher HeartQoL scores, both globally, as well as in emotional and physical dimensions, were associated with a lower severity of anxiety and depression assessed with the HADS: HADS-Anxiety (R Spearmann = −0.2909; *p* = 0.0001) and HADS-Depression (R Spearmann = −0.2583; *p* = 0.0002). Conclusions: The quality of life and functioning in chronic illness are connected with symptoms of depression and anxiety. When assessing the severity of the depression symptoms in relation to the individual aspects of functioning in chronic illness, the areas requiring supportive-educational intervention can be identified. The assessment of both functioning in a chronic disease and the severity of the depression symptoms should be included in a standard nursing diagnosis and further supportive and educational intervention.

## 1. Introduction

A chronic disease occurring in a person’s life is a stressor, disrupting every aspect of their life [1,2,3,4]. The co-occurrence of anxiety disorders and/or depression with chronic diseases is associated with poor functioning and a lower quality of life [5,6,7,8,9,10,11]. Depression is an independent risk factor for the development of cardiovascular diseases; furthermore, it worsens the prognosis in people already diagnosed with cardiovascular disorders [7,8,10,12,13]. 

Therefore, the need to assess daily functioning as well as the support from therapeutic teams in patients with chronic diseases has been suggested [14,15]. The complex relationships between anxiety and depression symptoms in relation to various aspects of patient functioning and quality of life undoubtedly merit multifaceted research. In our opinion, such justification exists especially in relation to patients at high cardiovascular risk, in whom atherosclerotic cardiovascular disease has not yet led to devastating consequences. However, to date, no studies have been conducted comprehensively analyzing the relationship between patient functioning and the occurrence of anxiety disorders and depression. The use of the validated FCIS questionnaire allows for a broad assessment of the patient’s functioning without the need to apply multiple tools. It allows for a quick diagnosis of the deficit areas and enables the planning of appropriate interventions [16].

This study aims to assess the relationship between functioning in chronic illness and quality of life with the prevalence of symptoms of depression and anxiety in patients without diagnosed atherosclerotic cardiovascular disease but with high cardiovascular risk. 

## 2. Materials and Methods

This observational cross-sectional study was conducted in 200 patients (aged 18–80 years), under the care of a general practitioner, who within 6–24 months prior to enrollment were diagnosed with hypertension. (ICD10: I10) and/or hypercholesterolemia (ICD-10: E78) and/or diabetes (E11) according to the current guidelines [17,18,19].

To the best of our knowledge, our study is the first to comprehensively assess the association between functioning in chronic disease and quality of life versus the prevalence of depression and anxiety symptoms in the cohort of high cardiovascular risk patients without diagnosed atherosclerotic cardiovascular disease. Due to the study design and the lack of evidence from previous studies to calculate sample power, the size of the study population was arbitrarily defined by the researchers. The consecutive patients were screened for study eligibility by 3 general practitioners cooperating with the investigators. In the next step, the initially selected candidates were re-evaluated by the investigators against the inclusion and exclusion criteria. The inclusion criteria were prior diagnosis of hypertension and/or hypercholesterolemia and/or diabetes. The exclusion criteria were prior diagnosed atherosclerotic cardiovascular disease, any requiring treatment disease, other than the cardiovascular risk factors defined as inclusion criteria, the inability to fill out the questionnaires independently, and the lack of informed consent to participate in this study. Each of the study participants gave their informed consent in this study in accordance with the principles of Good Clinical Practise and the requirements of the Declaration of Helsinki. This study was approved by the Ethics Committee of Nicolaus Copernicus University in Toruń, Collegium Medicum in Bydgoszcz (study approval reference number KB 586/2017). 

The study design was as follows:

1. Analysis of patients’ medical records—screening analysis conducted by primary care physicians to find eligible candidates for this study;
2. Interview with the patient conducted by the researcher (a qualified nurse or physician) to assess patients against the inclusion and exclusion criteria;
3. Providing the patient with information about this study and obtaining informed consent from the patient;
4. Conducting questionnaire surveys by the researcher, including the following:
  - Symptoms of anxiety and depression assessment with the Hospital Anxiety and Depression Scale (HADS);
  - Evaluation of quality of life with the Heart Quality of Life (HeartQoL) questionnaire;
  - Functioning in chronic illness assessment with the Functioning in Chronic Illness Scale (FCIS);
  - Patient survey.

The HADS (Hospital Anxiety and Depression Scale) is a tool that assesses the occurrence of symptoms of depression and anxiety. It consists of two subscales: HADS-Depression and HADS-Anxiety. The results obtained do not justify making a clinical diagnosis [20]. A score of 0–7 points corresponds to a normal level of anxiety/depression, 8–10 points to a borderline level, and 11–21 points to a high level, specific to the disease [21,22,23]. The HADS has been validated in Poland [24].

The HeartQoL (Heart Quality of Life) questionnaire is a standardized tool for assessing the quality of life of patients with cardiovascular diseases. It assesses the quality of life both globally (HeartQoL Global) and in two dimensions: emotional (HeartQoL Emotional) and physical (HeartQoL Physical). The questions are graded on a scale of 0 to 3 points. Higher results are associated with a better quality of life [25,26]. The HeartQoL questionnaire has been accepted for use in the EUROASPIRE V trial including a Polish arm [27].

The FCIS (Functioning in Chronic Illness Scale) is a validated tool that comprehensively assesses the functioning of patients with a division into 3 subscales: the impact of the disease on patient functioning (FCIS 1), the impact of the patient on the disease (FCIS 2), and the patient’s attitude towards the disease (FCIS 3). The higher the score in a given part of the questionnaire, the better the functioning of the patient in the studied area. Overall, a score below 78 is considered low, between 79 and 93 is medium, and above 94 is high [16]. The FCIS has been validated in Poland [16].

This study was limited to the analysis of the relationships between the studied variables and did not include an assessment of the therapeutic interventions outcome.

The statistical analysis was carried out using the Statistica 13.0 package (TIBCO Software Inc., Palo Alto, CA, USA). Continuous variables were presented as means with standard deviations. Categorical variables were expressed as the number and the percentage. The Shapiro–Wilk test demonstrated a non-normal distribution of the investigated continuous variables. Therefore, non-parametric tests were used for the statistical analysis. Comparisons between the groups were performed with the Kruskal–Wallis one-way analysis of variance. To assess the relationship between two continuous variables, Spearman’s rank correlation was used. The results were considered significant at *p* < 0.05. For the multivariate analysis, a multiple regression analysis was performed. The best models were identified using backward stepwise regression. The variables with no significant impact (*p* > 0.05) were removed one by one from the multivariate model according to the decreasing *p* value. 

## 3. Results

The HADS scores amounted to 4.34 ± 3.414 points for the HADS-Anxiety subscale and 3.20 ± 2.979 points for the HADS-Depression subscale. A high score was achieved by 3.0% and 6.5% of subjects, respectively, a borderline score in 13.0% and 6.5%, while the rest were within the normal range. The score indicative of functioning in chronic illness assessed with the FCIS was 98.32 ± 13.89 points. A low level of functioning was determined in 10% of the studied population, average in 25%, and high in as much as 65%.

The detailed characteristics of the study group are given in Table 1.

A multifactorial analysis (age, gender, hypertension, hypercholesterolemia, diabetes, FCIS global, and HeartQoL) showed that the independent predictors of a HADS-Anxiety score are HeartQoL Emotional and FCIS Global. These two factors explain 24.5% of the variability of a HADS-Anxiety score. In relation to HADS-Depression, the independent predictors are FCIS Global and HeartQoL Global. They explain 38.2% of the variability of a HADS-Depression score.

Better functioning in chronic illness (FCIS Global) was associated with less frequent symptoms of anxiety and depression based on the HADS: HADS-Anxiety (R Spearmann = −0.3969; *p* < 0.0001) and HADS-Depression (R Spearmann = −0.5884; *p* < 0.0001).

However, this relationship was expressed by the results obtained in two subscales of FCIS: assessing the impact of the disease on the patient (FCIS 1) and the patient’s attitude towards the disease (FCIS 3); but not in the FCIS 2 subscale, which determines the patient’s beliefs about their own impact on the course of the disease (Table 2).

Higher HeartQoL scores, both globally, as well as in emotional and physical dimensions, were associated with a lower severity of anxiety and depression assessed with the HADS: HADS-Anxiety (R Spearmann = −0.2909; *p* = 0.0001) and HADS-Depression (R Spearmann = −0.2583; *p* = 0.0002). These relationships were determined by the results of the HeartQoL Physical for HADS-Anxiety and both the HeartQoL Emotional and Physical for HADS-Depression (Table 3).

## 4. Discussion

In numerous observations, researchers point out that the feeling of anxiety and the occurrence of depression significantly affect both the prognosis and the functioning and quality of life of the patient [5,6,7,8,9,10,11]. 

To the best of our knowledge, our results are the first to show a correlation between the severity of depression and anxiety symptoms and the functioning in chronic illness.

In our study, the proportion of patients with symptoms of anxiety and depression was relatively low. A high severity of symptoms of depression was found in 3.0% of the subjects and of anxiety in 6.5% of the subjects, while borderline results reached 13.0% and 6.5%, respectively. The relatively low prevalence of depression and anxiety compared to other studies probably results from the exclusion of patients with diagnosed atherosclerotic cardiovascular disease.

A significantly higher proportion of patients with worsening symptoms of depression and anxiety can be found among patients with ischemic heart disease. After the onset of ischemic heart disease, depressive symptoms are diagnosed in 20–50% of patients, and severe depression develops in 15–20% of the cases [28,29].

It should be noted that the onset of depressive and anxiety symptoms after an acute cardiac event is an adaptive disorder that can be improved with comprehensive treatment [30]. It is equally important to implement preventive measures in people characterized by a high cardiovascular risk [31].

Many authors point to the need for a comprehensive assessment of the patient’s health and their adaptation to the new living conditions created by the onset of chronic illness [15,32,33,34,35,36,37]. The use of the FCIS (The Functioning in Chronic Illness Scale) allows for a multifaceted assessment of the patient’s functioning, which should be taken into account when planning therapeutic management [15,16,28,29,30,31,32,33,34,35,36,38,39,40,41].

We showed that lower levels of depression and anxiety were associated with better functioning in the disease. This relationship was determined by the results of the FCIS 1 and FCIS 3 subscales (i.e., the assessment of the impact of the disease on the patient’s life and the assessment of the patient’s attitudes towards the new life situation). Interestingly, we did not find that the patients’ beliefs about the possibility of influencing the course of the disease were associated with an increase in symptoms of depression and anxiety disorders. Studies suggest that the patient’s sense of agency has a positive effect on their process of coping with the disease, and thus on a better functioning and quality of life [42,43,44].

High-risk patients may be inadequately educated about the ability to influence their cardiovascular risk factors. This hypothesis can be confirmed by the results obtained by other authors [45,46].

In assessing the knowledge of risk factors for coronary disease, Buraczyński and Gotlib [45] indicate that patients do not identify hypertension, hypercholesterolemia, and diabetes as modifiable determinants of cardiovascular disease. Similar results were obtained by Dziedzic et al. [46].

The assessment of the quality of life of patients with chronic diseases is considered a necessary element in a comprehensive assessment of functioning in chronic illness. It is an additional source of information about the physical, mental, and social well-being of the patient [45,46,47,48]. Higher quality of life is associated with more effective control of risk factors, better functioning, and improved prognosis [32,39,40,47,48,49].

In our study, we found that increased depression and anxiety symptoms were associated with a lower quality of life, both emotionally and physically; furthermore, they contributed to obtaining lower scores in terms of functioning in chronic illness.

In summary, depressive and anxiety disorders are reflected in both the quality of life of the patients and their functioning in chronic disease [9,10,11,32,33,34,36,39,40,42,43,44,47,48]. The use of the FCIS questionnaire combined with the assessment of the anxiety and depression symptoms (HADS) allowed for the identification of new areas requiring nursing interventions. The efforts to improve the perception of the disease’s impact on the patient and to strengthen an optimistic outlook on the course of the disease may prove to be crucial in planning and conducting patient education.

Early diagnosis of the areas of functioning associated with the occurrence of anxiety and depressive symptoms may be the key to improving the adherence to the therapeutic recommendations [50,51,52,53,54,55].

The limitations of this study were the heterogeneity of the study population in terms of the time of diagnosis of the risk factors and the lack of precise data on the risk factors.

## 5. Conclusions

The quality of life and functioning in chronic illness are associated with symptoms of depression and anxiety.

The assessment of functioning in chronic illness allows for the diagnosis of areas requiring nursing interventions. Therapeutic education aimed at improving the patient’s perception of the disease’s impact on their health and enhancing optimism regarding the ability to influence the course of the disease can be crucial for reducing symptoms of anxiety and depression and improving patients’ quality of life.

## Figures and Tables

**Table 1:** Characteristics of the studied population.

Parameter	Variable	*n*	%
Age (mean ± SD)	51.6 ± 13.6		
Gender	Female	133	66.5
	Male	67	33.5
Diagnosed arterial hypertension	Yes	127	63.5
	No	73	36.5
Diagnosed hypercholesterolemia	Yes	90	45.0
	No	110	55.0
Diagnosed diabetes	Yes	41	20.5
	No	159	79.5
Functioning in chronic illness: FCIS Global	Low level	20	10.0
	Medium level	51	25.0
	High level	129	65.0
Functioning in chronic illness: FCIS Global	98.83 ± 13.89		
Quality of life: HeartQoL Global	2.65 ± 0.49		
Quality of life: HeartQoL Emotional	2.43 ± 0.66		
Quality of life: HeartQoL Physical	2.74 ± 0.50		
HADS-Anxiety	Normal	161	80.5
	Borderline	26	13.0
	Abnormal	13	6.5
HADS-Depression	Normal	181	90.5
	Borderline	13	6.5
	Abnormal	6	3.0
HADS-Anxiety	4.34 ± 3.41		
HADS-Depression	3.20 ± 2.97		

**Table 2:** Functioning in Chronic Illness Scale (FCIS) scores depending on the severity of the anxiety and depression assessed with the Hospital Anxiety and Depression Scale (HADS).

HADS		FCIS 1		FCIS 2		FCIS 3		FCIS Global	
ANXIETY	N	Mean ± SD	*p*	Mean ± SD	*p*	Mean ± SD	*p*	Mean ± SD	*p*
Normal	161	35.47 ± 5.22	<0.0001	31.21 ± 5.39	0.0336	34.45 ± 4.87	0.0080	101.12 ± 13.00	<0.0001
Borderline	26	31.35 ± 6.17		28.50 ± 4.49		31.19 ± 5.25		91.04 ± 12.06	
Abnormal	13	27.23 ± 8.72		29.54 ± 5.22		29.23 ± 7.05		86.00 ± 16.21	
**DEPRESSION**	**N**	**Mean ± SD**	***p***	**Mean ± SD**	***p***	**Mean ± SD**	***p***	**Mean ± SD**	***p***
Normal	181	35.39 ± 5.04	<0.0001	30.96 ± 5.36	0.0710	34.48 ± 4.63	<0.0001	100.82 ± 12.59	<0.0001
Borderline	13	27.69 ± 6.28		27.38 ± 4.07		27.31 ± 4.64		82.38 ± 11.80	
Abnormal	6	19.00 ± 4.27		31.67 ± 5.43		23.67 ± 6.89		74.33 ± 8.73	

**Table 3:** The Heart Quality of Life (HeartQoL) questionnaire scores depending on the severity of the anxiety and depression assessed with the Hospital Anxiety and Depression Scale (HADS).

HADS		HeartQoLEmotional		HeartQoL Physical		HeartQoL Global	
ANXIETY	N	Mean ± SD	*p*	Mean ± SD	*p*	Mean ± SD	*p*
Normal	161	2.54 ± 0.60	0.0001	2.78 ± 0.46	0.0018	2.71 ± 0.45	0.0002
Borderline	26	1.90 ± 0.72		2.63 ± 0.46		2.43 ± 0.44	
Abnormal	13	2.10 ± 0.73		2.41 ± 0.87		2.32 ± 0.78	
**DEPRESSION**	**N**	**Mean ± SD**	***p***	**Mean ± SD**	***p***	**Mean ± SD**	***p***
Normal	181	2.50 ± 0.60	0.0009	2.79 ± 0.43	0.0001	2.71 ± 0.42	0.0008
Borderline	13	1.94 ± 0.87		2.55 ± 0.40		2.38 ± 0.46	
Abnormal	6	1.46 ± 0.80		1.57 ± 1.05		1.54 ± 0.93	

## Data Availability

The data presented in this study are available from all authors.

## Public Involvement Statement

No public involvement in any aspect of this research.

## Guidelines and Standards Statement

This manuscript was drafted against The Strengthening 324 the Reporting of Observational Studies in Epidemiology (STROBE) Statement.
